# Performance of urine samples compared to cervical samples for detection of precancer lesions among HPV-positive women attending colposcopy clinic in Mexico City

**DOI:** 10.1007/s10552-024-01852-w

**Published:** 2024-02-18

**Authors:** Joacim Meneses-León, Sonia Hernández-Salazar, Leticia Torres-Ibarra, Rubí Hernández-López, Berenice Rivera-Paredez, Karina Robles-Rivera, Eduardo Lazcano-Ponce, Alba García-Vera, Mélany Godínez-Pérez, Leith León-Maldonado, Jorge Salmerón

**Affiliations:** 1 Facultad de Medicina, Centro de Investigación en Políticas, Población y Salud, Universidad Nacional Autónoma de México, Mexico, Mexico; 2 Centro de Investigación en Salud Poblacional, Instituto Nacional de Salud Pública, Cuernavaca, Morelos Mexico; 3 Oficina de Análisis del Plan de Salud, Subgerencia Técnica del Plan de Salud, Gerencia de Administración del Plan de Salud, Banco de México, Mexico, Mexico; 4 Dirección General, Instituto Nacional de Salud Pública, Cuernavaca, Morelos Mexico

**Keywords:** Cervical sample, Urine sample, Cancer screening, HPV

## Abstract

**Background:**

High-risk human papillomavirus (hrHPV) detection in self-collected urine samples (SeCUS) may be a promising alternative for cervical cancer screening because of its greater acceptability, as long as it can offer comparable sensitivity to clinician-collected cervical samples (CCoS) for detecting precancer lesions.

**Objective:**

To evaluate the performance of the SeCUS compared to that of the CCoS for cervical intraepithelial neoplasia grade 3 (CIN3) detection among hrHPV-positive women receiving colposcopy in Mexico City using different specific extended HPV typing procedures: HPV16/18, HPV16/18/35/39/68 or HPV16/18/35/39/68/31.

**Methods:**

From March 2017 to August 2018, 4,158 female users of the cervical cancer screening program at Tlalpan Sanitary Jurisdiction in Mexico City were invited to participate in the FRIDA-Tlalpan study. All participants provided ≥ 30 mL of SeCUS, and then a CCoS was obtained with Cervex-Brush®, which was used for hrHPV typing. Participants who tested positive for hrHPV in CCoS were referred for colposcopy for diagnostic confirmation, and all SeCUS of these women were also tested for hrHPV typing.

**Results:**

In total, 561 hrHPV-positive women were identified by CCoS via colposcopy, and 82.2% of the SeCUS of these women were also hrHPV positive. From both CCoS and SeCUS, 7 cases of CIN3 were detected. Considering HPV16/18 typing, CCoS and SeCUS detected 4 cases of CIN3, but after HPV16/18/35/39/68/31 extension typing, both CCoS and SeCUS detected all 7 of the CIN3 cases among the hrHPV-positive women.

**Conclusions:**

Using extended hrHPV typing based on HPV16/18/35/39/68/31, our results suggest that the performance of SeCUS may be equivalent to that of CCoS for detecting CIN3 lesions. Although our results are inconclusive, they support the hypothesis that SeCUS may be an attractive alternative worthy of further research.

**Supplementary Information:**

The online version contains supplementary material available at 10.1007/s10552-024-01852-w.

## Background

Cervical cancer is the fourth most common cancer in women and the fourth leading cause of cancer death worldwide, and more than 80% of these cases occur in low- and middle-income countries (LMICs) due to poor screening and delayed treatment. Globally, persistent human papillomavirus (HPV) infection contributes to more than 99% of HPV cases [1].

The ongoing improvement of diagnostic tools leading to high-throughput screening tests has changed the paradigm with the introduction of HPV genotyping as a primary screening test, and the efficacy of cervical cancer detection has increased compared to that of cytology-based screening [2]. Nevertheless, in Latin America, many barriers affecting the willingness of patients to undergo cervical cancer screening, including poor accessibility and poor availability of quality systems, have been reported [3]. In countries with many barriers to cervical cancer screening, 50% to more than 80% of women are not screened [4]. In contrast, in countries with well-organized screening programs, half of all potentially detectable carcinomas are found in women who have not attended screening programs [5].

Mexico has a low cervical cancer screening coverage of 59.7% [6]; one of the main reasons for this problem is the need to obtain a cervical sample for screening. The sample collection procedure is time-consuming and requires a trained clinician. HPV genotyping assays offer higher sensitivity for detection in different biological samples, making this assay an attractive tool for increasing the coverage of cervical cancer screening programs. As a screening method, HPV genotyping is also less invasive and uncomfortable considering the use of easy-to-self-collect vaginal or urine samples [7–9].

It has been reported that the cycle threshold value is higher for self-collected vaginal samples than for clinician-collected samples in routine primary HPV screening, and this approach offers a slightly lower sensitivity but a higher specificity for detecting CIN3 [10]. The use of self-collected urine samples (SeCUS) has been considered suitable as the primary collection method for cervical cancer due to its potential to encourage participation in screening in many settings where low acceptability for cervical sampling constitutes a barrier [11, 12]. A previous study reported that DNA obtained from a SeCUS for HPV testing, preserved in the presence of 40 mM ethylenediaminetetraacetic acid (EDTA) for up to 72 h before processing, corresponds well to that collected by CCoS, which has a kappa of 0.79 [11]. Several other studies have documented the accuracy of HPV testing on urine and vaginal self-collected samples compared to cervical samples, suggesting that self-collected samples are comparable in women receiving colposcopy; one of them had slightly higher sensitivities with similar specificities, and the other had similar sensitivities [13, 14].

Previous studies have documented greater acceptability of the SeCUS and an increase in the participation of women who do not regularly attend screening programs, and SeCUS is a low-cost procedure [8, 11, 15]. This finding supports the proposal that the SeCUS may be regarded as an acceptable alternative for collecting primary samples for cervical cancer screening, thereby increasing screening coverage for women who resist cervical sample collection [11]. Two meta-analyses based on screening studies with self-collected vaginal samples and SeCUS have reported that the detection of HPV is similar to that obtained with CCoS [4, 16]. While these results are promising, the evaluations of SeCUS come from studies under highly controlled collection and storage conditions [16], which are difficult to achieve in routine health service practices.

Additionally, although HPV testing may increase the effectiveness of cervical cancer screening programs, the majority of HPV infections may regress, and the infection will persist and lead to CIN2/CIN3 in only a small proportion of cases [17, 18]. This means that a high proportion of hrHPV-positive women do not require colposcopy, forcing public health planners to assess the best colposcopy referral algorithms for hrHPV-positive women. The most common currently used method is basic HPV16/18 typing, although extended typing (HPV16/18/35/39/68; HPV16/18/35/39/68/31) has also been suggested [19, 20].

Therefore, the objective of this study was to evaluate the performance of the SeCUS compared to that of the CCoS using extended specific hrHPV typing procedures, namely, HPV16/18, HPV16/18/35/39/68 or HPV16/18/35/39/68/31, for CIN3 detection among women from Mexico City under real conditions involving routine practice in an HPV-based cervical cancer screening program.

## Methods

### Study population

The study population included 4,158 women who were users of the cervical cancer screening program of the Sanitary Jurisdiction No. 8 in Tlalpan borough, Mexico City, who participated in the FRIDA-Tlalpan study [9] from March 2017 to August 2018. The protocol was approved by the Institutional Review Board of the National Institute of Public Health (INSP) (No. 1094). Informed consent was obtained from all the participants after the study procedures were explained. However, the analytical sample for the present analysis included all women with hrHPV-positive CCoS (*n* = 416) who underwent colposcopy for diagnostic confirmation.

### Procedures

Health personnel in each facility collected sociodemographic data and gynecological-obstetric history by administering a questionnaire to each woman who agreed to participate in the study.

#### Urine sample collection

All participants were asked to provide a SeCUS for hrHPV testing at the healthcare facility. For this purpose, the participants received a brochure with illustrated instructions and detailed verbal instructions from nurses on how to properly collect at least 30 mL of urine sample using a sterile container supplemented with a cell preservative [11]. To establish favorable storage conditions for obtaining DNA from urine and subsequent HPV detection, we performed sequential laboratory assays, considering EDTA as a DNA preservative. We evaluated the EDTA concentration, specimen storage temperature, time between urine collection and DNA extraction, and first-morning micturition versus convenience sample collection; the details of this procedure have been published elsewhere [11].

#### Cervical sample collection

A nurse, properly trained in cervical sampling, performed a pelvic exam to collect a cervical sample using Cervex-Brush® (Rovers Medical Devices). The sample was placed in a ThinPrep® vial (Roche®) for HPV determination, including hrHPV genotyping. The samples were preserved at room temperature until hrHPV testing was performed.

#### hrHPV detection

hrHPV detection via CCoS and SeCUS was performed on a BD Onclarity™ HPV assay (Becton, Dickinson and Company, DB Life Sciences-Diagnostic System; Sparks, MD) [4]. This assay uses the in vitro PCR test to individually identify HPV types 16, 18, 31, 45, 51, and 52 and groups 33/58, 56/59/66, and 35/39/68 in a single analysis. The SeCUS were processed consecutively, and the laboratory technicians were aware that all the samples were HPV+ according to the CCoS. The analysis was carried out following the manufacturer’s instructions, and the data were processed in the Molecular Diagnostic Laboratory of the INSP in Cuernavaca, Morelos, Mexico [9]. All SeCUSs of the hrHPV-positive CCoS patients attending colposcopy were also tested for hrHPV genotyping [11].

#### Diagnosis confirmation

Participants who tested positive for hrHPV in CCoS were referred for colposcopy for diagnostic confirmation and treatment if necessary. All women evaluated via colposcopy underwent a systematic collection of biopsies (at least one biopsy of the most abnormal zone of each quadrant) and an endocervical sample with an endocervical brush, regardless of colposcopy findings. These biopsies were processed in a private laboratory in Cuernavaca, Morelos. Histological evaluation was performed by a panel of pathologists who reported the diagnosis according to standard diagnostic criteria for precancerous lesions and cervical cancer [14].

### Statistical analysis

A descriptive analysis of the characteristics of the study population was performed considering all the screened women and all hrHPV-positive women who underwent colposcopy and had complete histological results. The prevalence of specific HPV types was estimated within each CCoS and SeCUS hrHPV-positive woman according to colposcopy.

To assess the performance of both CCoS and SeCUS for all women attending colposcopy because of hrHPV positivity, we quantified the number of tests performed, the rate of colposcopy referral, and the number of colposcopies performed to detect a CIN3 patient. The number of colposcopies per CIN3 detected is an indicator of the diagnostic efficiency of CCoS or SeCUS and was computed as the number of confirmed CIN3 lesions divided by the number of colposcopies performed [14]. These analyses were performed according to specific extended HPV typing scenarios: HPV16/18, HPV16/18/35/39/68, and HPV16/18/35/39/68/31.

Analyses were performed using Stata software version 14.0 (Stata Corp. LP: College Station, Tx).

## Results

A total of 4,158 women participated in our study and underwent the cervical cancer screening program; 13.5% of them (*n* = 561) were CCoS HPV-positive and referred for colposcopy, and only 416 attended colposcopy and completed diagnosis confirmation procedures. These women were slightly younger than all the screened women were (39.4 vs. 42.5 years, respectively), and 52.1% were married. The mean age at sexual debut was 18.6 years for all the screened women, and there was no difference among the women who underwent colposcopy. Over 40% of the women reported having 2–3 lifetime sexual partners (Table 1).

**Table 1:** Sociodemographic characteristics of study participants and analytical sample

	Screened women	hrHPV+ in cervical sample	Attended colposcopy with histological diagnosis
	*n* = 4,158	*n* = 561	*n* = 416
Age (years), mean (SD)	42.5 (10.0)	39.0 (9.8)	39.4 (9.9)
25–29	479 (11.5)	117 (20.9)	80 (19.2)
30–39	1,150 (27.7)	199 (35.5)	151 (36.3)
40–49	1,451 (34.9)	149 (26.6)	107 (25.7)
50–64	1,078 (25.9)	96 (17.1)	78 (18.8)
Marital status			
Married/common-law	2,877 (69.2)	292 (52.1)	215 (51.7)
Single	851 (20.5)	201 (35.8)	145 (34.9)
Divorced/separated/widowed	430 (10.3)	68 (12.1)	56 (13.4))
Age at sexual debut	18.6 (4.0)	18.1 (3.9)	18.2 (4.2)
Lifetime sexual partners			
1	1,519 (36.5)	101 (18.0)	77 (18.5)
2–3	1,784 (42.9)	263 (46.9)	189 (45.4)
> 4	855 (20.6)	197 (35.1)	150 (36.1)
hrHPV+			
CCoS	561 (13.5)	561 (100)	416 (100)
SeCUS^a^	–	492 (87.7)	367 (88.2)

^a^All paired SeCUS of HPV+ CCoS were tested for HPV with the BD Onclarity test. Out of the 561 SeCUS tested 492 were HPV+

**Fig. 1:**
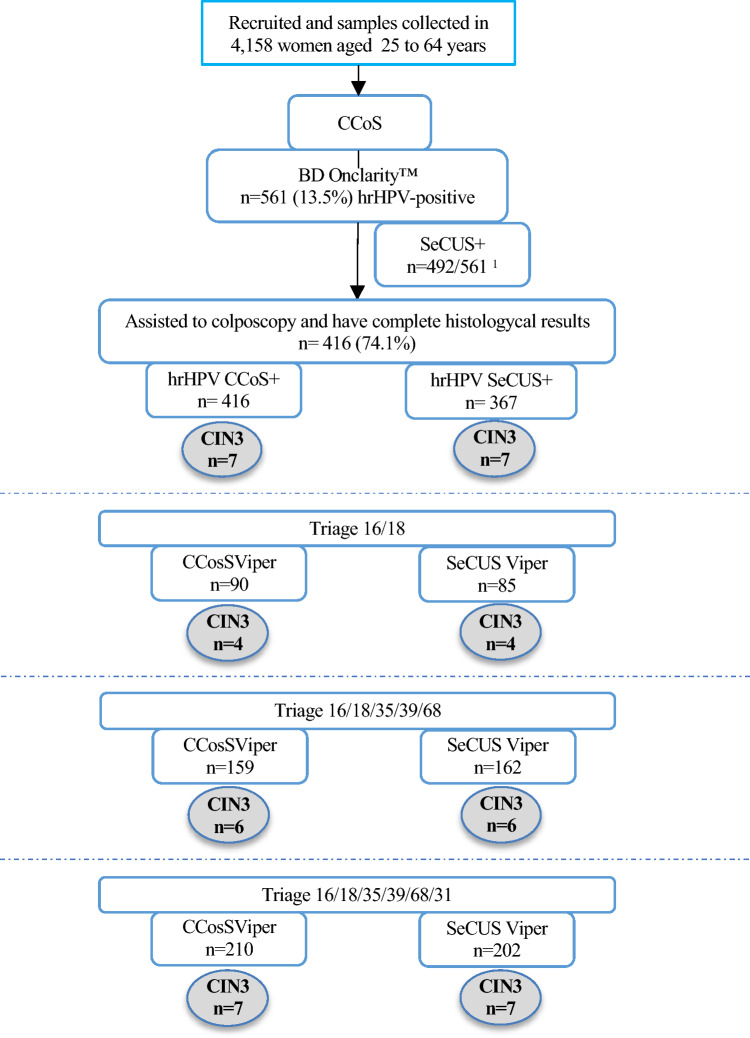
Flowchart of screening and different triage procedures. All paired SeCUS of HPV+ CCoS were tested for HPV with the BD Onclarity test. Out of the 561 SeCUS tested 492 were HPV+

We detected 7 CIN3 patients among the 416 women with hrHPV-positivity in the CCoS cohort. Among these patients, SeCUS also detected all 7 CIN3 patients (Fig. 1).

Using HPV16/18 as the colposcopy referral criterion, we found that the sensitivity of CIN3 detection was 57.1% for both CCoS and SeCUS. The sensitivity was 85.7% with the extended typing of HPV16/18/35/39/68, and 100% with the use of HPV16/18/35/39/68/31 (Fig. 1).

A total of 416 women with hrHPV-positivity among CCoS patients underwent colposcopy; however, only 88.2% of these women were hrHPV-positive by SeCUS. The prevalences of HPV16 and HPV18 were 16.1% and 6.0%, respectively, among CCoS hrHPV-positive women. Moreover, among the 367 SeCUS hrHPV-positive women, the prevalences of HPV16 and HPV18 were 17.4% and 6.3%, respectively (Table 2). In Table 2, we present the results of the cumulative prevalence, which we considered when selecting our hrHPV extended genotyping scenarios.

**Table 2:** Specific hrHPV types in participants with hrHPV positive results and among women attending colposcopy by collection method

	CCoS All hrHPV-positive Women *n* = 561		SeCUS All hrHPV-positive Women *n* = 492		CCoS hrHPV-positive Women attending colposcopy *n* = 416		SeCUS hrHPV-positive Women attending colposcopy *n* = 367	
hrHPV+	100%		87.7%		100%		88.2%	

	Cumulative prevalence^a^	Prevalence	Cumulative prevalence	Prevalence	Cumulative prevalence	Prevalence	Cumulative prevalence	Prevalence
HPV 16+	15.9	15.9	16.9	16.9	16.1	16.1	17.4	17.4
HPV 18+	21.0	5.9	22.2	5.9	21.6	6.0	23.2	6.3
HPV 35/39/68	38.5	19.4	43.3	25.4	38.2	18.8	44.1	25.9
HPV 31+	51.7	15.7	56.1	18.7	50.5	14.9	55.0	16.6
HPV 56/59/66	76.8	33.0	81.1	40.9	75.2	33.2	80.1	41.1
HPV 33/58	83.8	10.5	87.0	14.4	82.0	9.6	85.6	13.1
HPV 51+	91.1	11.6	92.9	14.4	89.9	11.8	90.1	14.7
HPV 52+	97.5	12.3	97.7	14.8	97.1	13.5	97.8	16.3
HPV 45+	100	5.5	100	7.3	100	4.8	100	6.5

^a^Cumulative prevalence: Refers to the total prevalence, adding the prevalence of each specific types detected in hrHPV detection

We also evaluated the number of colposcopy procedures for detecting CIN3 cases and found that the number of colposcopy procedures was similar between CCoS and SeCUS. With the HPV16/18 referring strategy in CCoS, 23 procedures are needed, while in SeCUS, 21 procedures are needed. However, with extended typing, which included HPV16/18/35/39/68/31 CCoS, 30 patients were required to be diagnosed with CIN3, while SeCUS 29 was needed (Table 3).

**Table 3:** Performance of CCoS and SeCUS with different HPV extended typing alternatives to detect CIN3

Sample		HPV-based screening^a^	Women with HPV+ results^b^	Women with complete histological diagnosis^c^	CIN3 cases detected^d^	Colposcopy procedures to detect a CIN3 case^e^
CCoS	All hrHPV	4,158	561	416	7	59
SeCUS	All hrHPV	4,158	492	367	7	52
	Extended typing					
CcoS	HPV16/18		118 (21.0%)	90	4	23
SeCUS	HPV16/18		109 (22.2%)	85	4	21
CcoS	HPV16/18/35/39/68		216 (38.5%)	159	6	27
SeCUS	HPV16/18/35/39/68		213 (43.3%)	162	6	27
CcoS	HPV16/18/35/39/68/31		290 (51.7%)	210	7	30
SeCUS	HPV16/18/35/39/68/31		276 (56.1%)	202	7	29

^a^All study participants with HPV screening

^b^Participants with HPV+ result, considering that all paired SeCUS of HPV+ CCoS were tested for HPV with the BD Onclarity test. Out of the 561 SeCUS tested 492 were HPV+

^c^The numbers in this column are considering only those women that attended colposcopy and completed diagnosis confirmation procedure, representing only 74.1% of all HPV+ CCoS

^d^Number of CIN3 cases among women with complete histological diagnosis

^e^This is just de rate of number of CIN3 cases detected divided by colposcopy procedures performed in each extended typing group

## Discussion

We observed that under the scenario of hrHPV16/18, 4 CIN3 cases were detected by CCoS, while the same 4 CIN3 cases were detected by SeCUS, with a sensitivity of 57.1% for CIN3 in both CCoS and SeCUS. We tested different specific HPV typing strategies, such as adding HPV 35/39/68 to CCoS and SeCUS, and detected 6 CIN3 patients, increasing the sensitivity to 85.7%. Our final reference strategy considered HPV16/18/35/39/68/31 with a sensitivity of 100%, detecting all 7 patients in the COoS and SeCUS cohorts. The different hrHPV extended genotyping regimens significantly reduced the number of colposcopies needed to detect CIN3, which was even slightly greater when using SeCUS.

New guidelines propose the concept of “equal management for equal risk” regarding colposcopy referral, considering that this recommendation is very important for identifying specific HPV types as an efficient strategy for reducing referral for diagnostic confirmation procedures [19]. To our knowledge, this is the first analysis evaluating extended specific HPV genotyping strategies in SeCUS without considering cytology. Our results suggest the possibility of skipping cytology, as we also analyzed a scenario with HPV typing and cytology results from CCoS, and cytology did not reveal any additional CIN3. In this sense, extended genotyping of hrHPV in SeCUS as a referral strategy could be useful for cervical cancer screening programs based on self-sampling, where it is difficult to perform cytological triage on the same sample [5, 21].

Despite the improvements in cervical cancer screening programs in countries such as Mexico, many women still miss the opportunity to detect precancerous lesions by not receiving preventive medicine services. For this reason, simpler screening alternatives, such as SeCUS, must be offered. Screening for HPV infection in SeCUS patients has shown greater acceptability among women, which could translate into greater participation in screening programs [5, 8, 10]. The findings of this work may be useful in screening Mexican women, and the results may support the recommendation of the use of the SeCUS for cervical cancer screening.

hrHPV-positive SeCUSs were not sent for colposcopy; only CCoS was used, which prevents us from estimating the specificity of SeCUS. Although this is an important limitation, this initial analysis allows us to establish the equivalence of the sensitivities. The small sample size of the analytical sample is an additional limitation of this study; however, it is an initial approach to the use of HPV extended genotyping in SeCUS as an appealing strategy for further studies. The present study has many strengths, such as the use of real conditions during a cervical cancer screening program in Mexico and the use of a less expensive strategy in LMICs; moreover, we can imagine that this method is applicable to different settings. Additionally, our results obtained using SeCUS with ethylenediaminetetraacetic acid (EDTA) were equivalent to the results obtained for cervical samples, confirming—as previously reported in the literature—the validity of this device for collection using it as a preservative medium [10, 20].

Overall, the results of this analysis of extended hrHPV typing suggest that the performance of the SeCUS may be equivalent to that of the CCoS in detecting CIN3 lesions. Our findings are not conclusive but support the hypothesis that the SeCUS may be an appealing alternative for HPV testing at real-life screening visits.

### Supplementary Information

Below is the link to the electronic supplementary material.

Supplementary file1 (DOCX 19 KB)

## Data Availability

The datasets generated during and/or analyzed during the current study are not publicly available due to confidentiality issues but are available from the corresponding author upon reasonable request.
