# Supplementary material for: Performance of urine samples compared to cervical samples for detection of precancer lesions among HPV-positive women attending colposcopy clinic in Mexico City
Source: Cancer Causes Control. 2024 Feb 18;35(6):935–42. doi: 10.1007/s10552-024-01852-w (PMC11129980; doi:10.1007/s10552-024-01852-w)
Supplement: Supplementary file 1 — Supplementary file1 (DOCX 19 KB) [file 10552_2024_1852_MOESM1_ESM.docx]

| **Supplementary table 1. Performance of CCoS and SeCUS with different HPV extended typing alternatives to detect cervical lesions** | | | | | | | |
| --- | --- | --- | --- | --- | --- | --- | --- |
| Sample |  | HPV-based screening | Women with positive results | Women with complete histological diagnosis | CIN1 cases detected | CIN2  cases detected | Colposcopy procedures to detect a CIN case |
| CCoS | All hrHPV | 4,158 | 561 | 416 | 63 | 12 | 35 |
| SeCUS | All hrHPV | 4,158 | 492 | 367 | 58 | 9 | 41 |
|  | **Extended typing** |  |  |  |  |  |  |
| CCoS | HPV16/18 |  | 118 | 90 | 17 | 8 | 11 |
| SeCUS | HPV16/18 |  | 109 | 85 | 19 | 5 | 17 |
| CCoS | HPV16/18/35/39/68 |  | 216 | 159 | 29 | 8 | 20 |
| SeCUS | HPV16/18/35/39/68 |  | 213 | 162 | 31 | 5 | 32 |
| CCoS | HPV16/18/35/39/68/31 |  | 290 | 210 | 32 | 9 | 23 |
| SeCUS | HPV16/18/35/39/68/31 |  | 276 | 202 | 35 | 6 | 34 |
|  |  | | | | | | |
